# Diagnostic value of fibulin-3 for malignant pleural mesothelioma: A systematic review and meta-analysis

**DOI:** 10.18632/oncotarget.12707

**Published:** 2016-10-17

**Authors:** Ran Ren, Pengpeng Yin, Yan Zhang, Jianyun Zhou, Yixing Zhou, Rufu Xu, Hai Lin, Chunji Huang

**Affiliations:** ^1^ Division of Scientific Research, Xinqiao Hospital, Third Military Medical University, Chongqing, 400037, China; ^2^ Department of Scientific Research, Third Military Medical University, Chongqing, 400038, China

**Keywords:** Fibulin-3, malignant pleural mesothelioma, diagnostic value, meta-analysis, systematic review

## Abstract

**Background:**

Several studies have investigated the diagnostic value of fibulin-3 for malignant pleural mesothelioma (MPM), but the results were various. Therefore, we performed a systematic review and meta-analysis to evaluate the diagnostic value of fibulin-3 for MPM.

**Results:**

Eight studies were included in this work. The overall sensitivity of blood fibulin-3 were 0.87 (95% CI, 0.58 – 0.97) and 0.89 (95% CI, 0.77 – 0.95), respectively. The overall sensitivity and specificity of PF fibulin-3 for MPM were 0.73 (95% CI, 0.54 – 0.86) and 0.80 (95% CI, 0.60 – 0.91), respectively. The area under curve of blood and pleural effusion (PF) Fibulin-3 were 0.94 (95% CI, 0.91 – 0.96) 0.83 (95% CI, 0.79 – 0.86), respectively.

**Methods:**

PubMed and EMBASE databases were searched up to July 29, 2016 to verify studies investigating the diagnostic value of fibulin-3 for MPM. The quality of eligible studies was assessed using the revised Quality Assessment for Studies of Diagnostic Accuracy tool (QUADAS-2). The overall sensitivity and specificity were pooled using a bivariate model.

**Conclusion:**

Fibuoin-3 is a useful diagnostic marker for MPM.

## INTRODUCTION

Malignant pleural mesothelioma (MPM) is one of the most common cancers in asbestos-exposed individuals around the world [1, 2]. Timely and accurate diagnosis of MPM can improve the outcomes of patients [3]. Currently, the diagnosis of MPM mainly relies on pleural biopsy, which is invasive and the sample error is a problem [4, 5]. Therefore, developing non-invasive biomarkers for MPM diagnosis is of great value [6]. During past decades, many circulating biomarkers for MPM has been developed, such as soluble mesothelin-related peptides (SMRP) [7, 8] and osteopontin [9, 10]. However, the sensitivity and specificity of theses biomarkers are modest. Therefore, it is valuable to explore novel biomarkers that can improve the diagnostic value of the traditional biomarkers or replace them.

Fibulin-3 is a secreted glycoprotein that plays an important role in the regulation of cell migration and proliferation [11]. During past years, many studies have revealed that fibulin-3, either in blood or pleural effusion (PF), is a potential diagnostic biomarker for MPM [6]. However, the results from these studies were heterogeneous. Therefore, we performed a systematic review and meta-analysis to investigate the diagnostic value of fibulin-3 for MPM.

## RESULTS

### Summary of eligible studies

**Figure 1:**
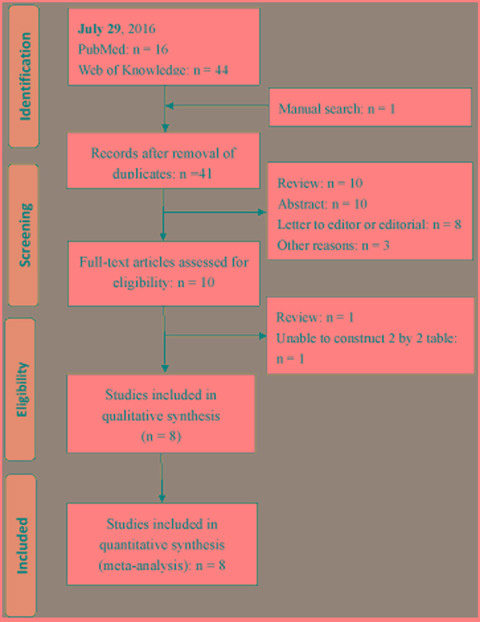
Flow chart depicting the literature search and study selection process

Eight studies were included in present systematic review and meta-analysis [12–19]. A flowchart depicting study selection is shown in Figure 1. Summary of eligible studies is listed in Table 1. Eight studies investigated the diagnostic value of blood (three used serum [15, 17, 18] and five used plasma [12–14, 16, 19]) fibulin-3 for MPM and five studies investigated the diagnostic value of PF fibulin-3. The study performed by Pass et.al. [12] contained two study cohorts and thus is regarded as two independent studies. The sample sizes ranged from 36 to 228. Components of control in eligible studies were various, including asbestos-exposed persons [12, 13, 18, 19], patients with pleural effusion [13, 16, 19] or metastatic pleural malignancy [15], healthy controls [17] or patients with extrapleural pneumonectomy [14]. One study did not report reference standard used for MPM diagnosis [12], and one study [13] used biopsy and follow-up to diagnose MPM. The remaining studies used biopsy to as reference standard. One study [14] was retrospective design and two [15, 19] did not report the type of design; and the remaining studies were prospective design.

**Table 1:** Summary of included studies

Author	Year	Country	N	Component of control	Reference	Specimen	Design
Blood							
Pass [12], Detroit cohort	2012	USA	228	AEPs	NR	Plasma	Prospective
Pass [12], Toronto cohort	2012	USA	144	AEPs	NR	Plasma	Prospective
Creaney [13]	2014	Australia	202	PFs; AEPs	Biopsy and follow-up	Plasma	Prospective
Kirschner [14]	2015	Switzerland	130	Patients with extrapleural pneumonectomy or undergoing cardiac or aortic surgery for CAD or aortic disease, pleural plaques or pleuritis	Biopsy	Plasma	Retrospective
Agha [15]	2014	Egypt	36	Metastatic pleural malignancy	Biopsy	Serum	Unknown
Elgazzar [16]	2014	Egypt	60	Malignant PFs	Biopsy	Plasma	Prospective
Kaya [17]	2015	Turkey	83	Healthy controls	Biopsy	Serum	Prospective
Demir [18]	2016	Turkey	90	AEPs	Biopsy	Serum	Prospective
Napolitano [19]	2016	USA and UK	80	Benign PFs; AEPs	Biopsy	Plasma	Unknown
PF							
Pass [12], Detroit cohort	2012	USA	167	Patients with PF	NR	PF	Prospective
Creaney [13]	2014	Australia	174	Patients with PF	Biopsy and follow-up	PF	Prospective
Kirschner [14]	2015	Australia	90	Patients with PF	Biopsy	PF	Retrospective
Agha [15]	2014	Egypt	36	Patients with PF	Biopsy	PF	Unknown
Elgazzar [16]	2014	Egypt	60	Patients with PF	Biopsy	PF	Prospective

N, sample size; PF, pleural effusion; CAD, coronary artery disease; AEP, asbestos-exposed person; NR, not reported.

### Quality assessment of eligible studies

Quality assessment of eligible studies is listed in Table 2. The patient selection domain of all eligible studies was labeled as high because lacking of uniform including and excluding criteria, and the study cohort in each eligible studies was not enrolled consecutively. Index domain in all eligible studies, except one [13], was labeled as unknown because whether index test was performed in a blind manner was not reported. Reference standard domain in one study [12] was labeled as unknown because the diagnostic criteria for MPM was not reported. Flow and timing domain of four studies was labeled as high because of the differential verification bias [17, 18], partial verification bias [12] or disease progression bias [13].

**Table 2:** Quality assessment of eligible studies

Study	Risk of bias				Applicability concerns		
	Patient selection	Index test	Reference standard	Flow and timing	Patient selection	Index test	Reference standard
Serum							
Pass [12], Detroit cohort	High	Unknown	Unknown	High	Low	Low	Unknown
Pass [12], Toronto cohort	High	Unknown	Unknown	High	Low	Low	Unknown
Creaney [13]	High	Low	Low	High	Low	Low	Low
Kirschner [14]	High	Unknown	Low	Low	High	Low	Low
Agha [15]	High	Unknown	Low	Low	Low	Low	Low
Elgazzar [16]	High	Unknown	Low	Low	Low	Low	Low
Kaya [17]	High	Unknown	Low	High	High	Low	Low
Demir [18]	High	Unknown	Low	High	Low	Low	Low
Napolitano [19]	High	Unknown	Low	Low	Low	Low	Low
PF							
Pass [12]	High	High	Unknown	High	Low	Low	Unknown
Creaney [13]	High	Low	Low	High	Low	Low	Low
Kirschner [14]	High	Unknown	Low	Low	Low	Low	Low
Agha [15]	High	Unknown	Low	Low	Low	Low	Low
Elgazzar [16]	High	Unknown	Low	Low	Low	Low	Low

### Diagnostic value of fibulin-3 for MPM

Table 3 lists the diagnostic value of fibulin-3 in each eligible studies. All studies used ELISA to determine fibulin-3.

**Table 3:** Diagnostic value of fibulin-3 in eligible publications

Author	Test method	MPM/Control	Cut-offs	TP	FN	FP	TN
Blood							
Pass [12], Detroit cohort	ELISA	92/136	52.8 ng/ml	66	26	0	136
Pass [12], Toronto cohort	ELISA	48/96	28.96 ng/ml	35	13	11	85
Creaney [13]	ELISA	82/120	29 ng/ml	39	43	35	85
Kirschner [14]	ELISA	84/56	29 ng/ml	11	73	4	52
Agha [15]	ELISA	25/11	66.5 ng/ml	22	3	2	9
Elgazzar [16]	ELISA	30/30	54.3 ng/ml	30	0	1	29
Kaya [17]	ELISA	43/40	30.1 ng/ml	42	1	5	35
Demir [18]	ELISA	42/48	51.41 ng/ml	37	5	16	32
Napolitano [19]	ELISA	22/58	29 ng/ml	22	0	15	43
PF							
Pass [12]	ELISA	74/93	346.01 ng/ml	62	12	11	82
Creaney [13]	ELISA	103/71	346 ng/ml	61	42	34	37
Kirschner [14]	ELISA	30/60	346 ng/ml	14	22	22	38
Agha [15]	ELISA	25/11	150 ng/ml	18	7	2	9
Elgazzar [16]	ELISA	30/30	520 ng/ml	27	3	1	29

PF, pleural effusion; ELISA, enzyme-linked immunosorbent assay; TP, true positive; TN, true negative; FP, false positive; FN, false negative.

**Figure 2:**
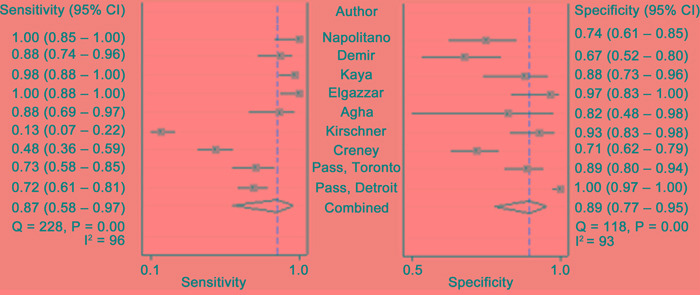
Forest plots estimating the sensitivity and specificity of blood fibulin-3 for MPM Each point represents the sensitivity and specificity of each eligible studs and error bars are 95% CIs.

Figure 2 is a forest plot for blood fibulin-3. The overall sensitivity and specificity of blood fibulin-3 for MPM were 0.87 (95% CI, 0.58 – 0.97) and 0.89 (95% CI, 0.77 – 0.95), respectively. Significant heterogeneity was observed for both sensitivity and specificity, with *I^2^* of 96 and 93 respectively. Table 4 lists the results of subgroup analysis. Type of data collection (prospective or others), matrix used for fibulin-3 measurement (serum or plasma) and the components of controls (asbestos-exposed individual only or others), were not the sources of heterogeneity.

**Table 4:** Diagnostic accuracy of blood fibulin-3 for MPM

	Cohorts	Sensitivity (95% CI)	Specificity (95% CI)
Blood			
All	9	0.87 (0.58 – 0.97)	0.89 (0.77 – 0.95)
Specimen			
Serum	3	0.94 (0.80 – 1.00)	0.80 (0.58 – 1.00)
Plasma	6	0.79 (0.52 – 1.00)	0.92 (0.84 – 1.00)
Design			
Prospective	6	0.88 (0.70 – 1.00)	0.91 (0.81 – 1.00)
Retrospective or unknown	3	0.81 (0.42 – 1.00)	0.86 (0.67 – 1.00)
Control			
AEP only	3	0.79 (0.40 – 1.00)	0.93 (0.83 – 1.00)
Others	6	0.90 (0.72 – 1.00)	0.87 (0.74 – 0.99)

AEP, asbestos-exposed person; CI, confidence interval.

The overall sensitivity and specificity of PF fibulin-3 for MPM were 0.73 (95% CI, 0.54 – 0.86) and 0.80 (95% CI, 0.60 – 0.91), respectively. The *I^2^* for sensitivity and specificity were 91 and 92 respectively, indicating that great heterogeneity was exist among eligible studies.

**Figure 3:**
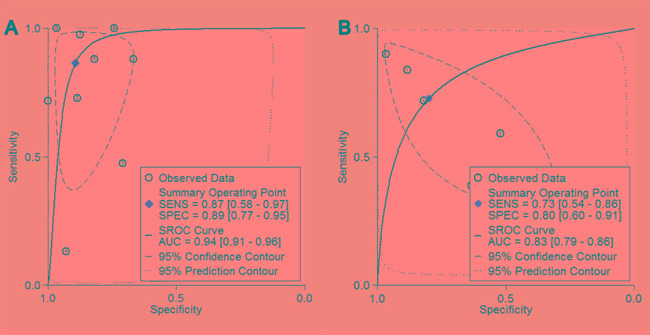
Summary receiver operating characteristic curves for overall diagnostic accuracy of blood fibulin-3

The AUCs for sROC of blood and PF fibulin-3 were 0.94 (95% CI, 0.91 – 0.96) and 0.83 (95% CI, 0.79 – 0.86), respectively (Figure 3). The diagnostic odds ratios (DORs) for blood and PF fibulin-3 were 53 (95% CI, 10 – 289) and 11 (95% CI, 2 – 59), respectively.

### Publication bias

**Figure 4:**
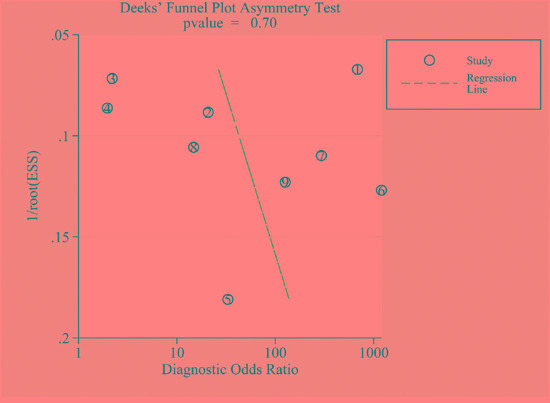
A Funnel plot assessing publication bias

Figure 4 shows a funnel plot for the eligible studies. Obvious symmetry was observed, indicating publication bias is insignificant (*P*= 0.70).

## DISCUSSION

To the best of our knowledge, this is the first meta-analysis investigating the diagnostic value of blood and PF fubulin-3 for MPM. The major findings of present study are as follows. First, both blood and PF fibulin-3 were useful diagnostic markers for MPM. Second, the available studies have some design weakness and further well-designed studies are needed to rigorously evaluate the diagnostic value of fibulin-3. Third, there was no publication bias among all available studies, indicating that the results of present work are reliable.

To present, many diagnostic biomarkers for MPM has been developed. Among these biomarkers, SMRP and osteopontin are the most promising ones [12, 20]. Evidence from meta-analysis revealed that the diagnostic sensitivity and specificity for SMRP were 0.61 and 0.87, respectively [7]. For osteopontin, a meta-analysis also revealed that its sensitivity and specificity were 0.65 and 0.81, respectively [10]. Our study revealed that the diagnostic specificity of blood fibulin-3 was 0.89, which was comparable to those of SMRP and osteopontin. However, the sensitivity of blood fibulin-3 was 0.87, which was obviously higher than those of SMRP and osteopontin. Therefore, blood fibulin-3 represents a promising diagnostic marker for MPM, and it may have a potential to replace SMRP or osteopontin in MPM diagnosis. Further studies designed in a head-to-head comparison manner are needed to clarify whether the diagnostic value of fibulin-3 is superior to those of SMRP and osteopontin.

AUC of sROC is an index determining overall diagnostic value of a test [21, 22]. Our study revealed that the AUCs for blood and PF fibuin-3 were 0.94 and 0.83, respectively, indicating that both PF and blood fibulin-3 are useful for MPM diagnosis. Besides, previous meta-analysis also show that the AUCs for SMRP and osteopontin were 0.81 and 0.83, respectively, also supporting our hypothesis that the diagnostic value of fibulin-3 is superior to those of SMRP and osteopontin.

The sources of PF fibulin-3 are largely unknown. Three studies have investigated the correlation between circulating and PF fibulin-3 [12, 13, 15], and two failed to observe a positive correlation between circulating and PF fibulin-3 [12, 13]. These results indicate that PF fibulin-3 is not derived from circulating fibulin-3, and measuring fibulin-3 in PF may yield additional diagnostic value. However, in a study performed by Agha et al. [15], there was a good relationship between serum and PF fibulin-3. Reasons for inconsistent finding across these three studies are unknown. Further studies are needed to elucidate the basis of the discrepancy.

An exploration of the sources for heterogeneity, rather than pooling the results of all eligible studies, is an important goal of meta-analysis. Because great heterogeneity was observed across all eligible studies investigating diagnostic value of blood fibulin-3 for MPM, we performed a subgroup analysis to explore the sources of heterogeneity. We found that some of the design characteristics, including type of data collection, matrix used for fibulin-3 measurement and the components of controls, were not the sources of heterogeneity. Future studies with more eligible studies are needed to explore the sources of heterogeneity.

To facilitate more well-designed future studies on this topic, some of the methodological weakness of the available studies should be noted. The major design weakness of available studies was two-gate design [23], which can result participant selection bias. The subjects in all studies were not enrolled according to a pre-designed inclusion and exclusion criteria. That means, the subjects enrolled in these studies may not be representative of the target population in whom MPM is suspected. Therefore, the conclusions of available studies should be interpreted with caution.

Taken together, present study indicated that fibulin-3 was a useful diagnostic marker for MPM. Due to the small number of eligible studies, and all of the eligible studies have higher risk for subject selection, further well-designed studies are needed to rigorously evaluate the diagnostic value of fibulin-3 for MPM.

## MATERIALS AND METHODS

### Database and literature retrieve

This systematic review and meta-analysis was performed in accordance with preferred reporting items for systematic reviews and meta-analyses (PRISMA) guideline [24]. Two investigator independently searched PubMed and EMBASE for potential eligible studies. The last search date is July 29, 2016. The searched algorithm used for searching PubMed was “(Fibulin-3 OR EFEMP1 protein, human[nm] OR “Fibulin 3”) AND mesothelioma”. Similar search strategy was used for searching EMBASE. A manual search was also performed by reviewing references listed at the end of retrieved publications.

### Inclusion and exclusion criteria

Inclusion criteria of this systematic review and meta-analysis were: (1) studies investigating the diagnostic value of fibulin-3, either in blood or PF, for MPM; (2) A 2 by 2 table can be constructed using sensitivity, specificity and sample size reported, or using data presented in the scatter plot. Conference abstracts and animal studies were excluded. Studies with sample size less than 10 were excluded because studies with small sample sizes can yield bias.

The study selection process was performed by two independent investigators. In the first round, the titles and abstracts of retrieved publications were screened and the irrelevant studies were excluded. In the second round, a full text reviewing was performed to select eligible studies for the remaining studies. Disagreements were resolved by consensus or full text review.

### Data extraction and quality assessment

Two investigators independently extracted data from eligible studies. Following data were extracted: sample size, publication year, sources of participants, components of control, reference standard used for MPM diagnosis, type of design (prospective or retrospective) and fibulin-3 measurement methods. For each eligible studies, a 2 by 2 table, which consisted of true positive (TP), false negative (FN), false positive (FP) and true negative (TN), was constructed.

The revised Quality Assessment for Studies of Diagnostic Accuracy tool (QUADAS-2) [25] was used to assess the quality of eligible studies. Any disagreement in quality assessment was resolved by consensus. The corresponding authors of the eligible studies were not contacted for unknown information regarding study design.

### Statistical analysis

The overall sensitivity and specificity of fibulin-3 for MPM diagnosis were pooled using a bivariate model [26]. The summary receiver operating characteristic (sROC) curve was constructed to depict the overall diagnostic value of fibulin-3 [27]. The funnel plots and the Deeks's test were used to test publication bias [28]. All analyses were performed in STATA 13.0 (Stata Corp LP, College Station, TX) and the midas command was used for all statistical analyses.

## References

[1] Soeberg MJ, Leigh J, Driscoll T, Armstrong B, Young JM, van Zandwijk N (2016). Incidence and survival trends for malignant pleural and peritoneal mesothelioma, Australia, 1982-2009. Occup Environ Med.

[2] Ismail-Khan R, Robinson LA, Williams CC, Garrett CR, Bepler G, Simon GR (2006). Malignant pleural mesothelioma: a comprehensive review. Cancer Control.

[3] van Zandwijk N, Clarke C, Henderson D, Musk AW, Fong K, Nowak A, Loneragan R, McCaughan B, Boyer M, Feigen M, Currow D, Schofield P, Nick Pavlakis BI (2013). Guidelines for the diagnosis and treatment of malignant pleural mesothelioma. J Thorac Dis.

[4] Zhang W, Wu X, Wu L, Zhang W, Zhao X (2015). Advances in the diagnosis, treatment and prognosis of malignant pleural mesothelioma. Ann Transl Med.

[5] Wald O, Sugarbaker DJ (2016). Perspective on malignant pleural mesothelioma diagnosis and treatment. Ann Transl Med.

[6] Panou V, Vyberg M, Weinreich UM, Meristoudis C, Falkmer UG, Roe OD (2015). The established and future biomarkers of malignant pleural mesothelioma. Cancer TreatRev.

[7] Cui A, Jin XG, Zhai K, Tong ZH, Shi HZ (2014). Diagnostic values of soluble mesothelin-related peptides for malignant pleural mesothelioma: updated meta-analysis. BMJ Open.

[8] Hollevoet K, Reitsma JB, Creaney J, Grigoriu BD, Robinson BW, Scherpereel A, Cristaudo A, Pass HI, Nackaerts K, Rodriguez Portal JA, Schneider J, Muley T, Di Serio F (2012). Serum mesothelin for diagnosing malignant pleural mesothelioma: an individual patient data meta-analysis. J Clin Oncol.

[9] Lin H, Shen YC, Long HY, Wang H, Luo ZY, Wei ZX, Hu SQ, Wen FQ (2014). Performance of osteopontin in the diagnosis of malignant pleural mesothelioma: a meta-analysis. Int J Clin Exp Med.

[10] Hu ZD, Liu XF, Liu XC, Ding CM, Hu CJ (2014). Diagnostic accuracy of osteopontin for malignant pleural mesothelioma: a systematic review and meta-analysis. Clin Chim Acta.

[11] Creaney J, Dick IM, Robinson BW (2015). Comparison of mesothelin and fibulin-3 in pleural fluid and serum as markers in malignant mesothelioma. Curr Opin Pulm Med.

[12] Pass HI, Levin SM, Harbut MR, Melamed J, Chiriboga L, Donington J, Huflejt M, Carbone M, Chia D, Goodglick L, Goodman GE, Thornquist MD, Liu G (2012). Fibulin-3 as a blood and effusion biomarker for pleural mesothelioma. N Engl J Med.

[13] Creaney J, Dick IM, Meniawy TM, Leong SL, Leon JS, Demelker Y, Segal A, Musk AW, Lee YC, Skates SJ, Nowak AK, Robinson BW (2014). Comparison of fibulin-3 and mesothelin as markers in malignant mesothelioma. Thorax.

[14] Kirschner MB, Pulford E, Hoda MA, Rozsas A, Griggs K, Cheng YY, Edelman JJ, Kao SC, Hyland R, Dong Y, Laszlo V, Klikovits T, Vallely MP (2015). Fibulin-3 levels in malignant pleural mesothelioma are associated with prognosis but not diagnosis. Br J Cancer.

[15] Agha M, El-habashy M, El-Shazly R (2014). Role of fibulin-3 in the diagnosis of malignant mesothelioma. Egypt J Chest Dis Tuberc.

[16] Elgazzar AEM, Embarak S, Refat AM, Bakry A, Mokhtar A (2014). Value of plasma and pleural effusion fibulin-3 levels in the diagnosis of malignant pleural mesothelioma effusions. Egypt J Chest Dis Tuberc.

[17] Kaya H, Demir M, Taylan M, Sezgi C, Tanrikulu AC, Yilmaz S, Bayram M, Kaplan I, Senyigit A (2015). Fibulin-3 as a diagnostic biomarker in patients with malignant mesothelioma. Asian Pac J Cancer Prev.

[18] Demir M, Kaya H, Taylan M, Ekinci A, Yilmaz S, Teke F, Sezgi C, Tanrikulu AC, Meteroglu F, Senyigit A (2016). Evaluation of New Biomarkers in the Prediction of Malignant Mesothelioma in Subjects with Environmental Asbestos Exposure. Lung.

[19] Napolitano A, Antoine DJ, Pellegrini L, Baumann F, Pagano I, Pastorino S, Goparaju CM, Prokrym K, Canino C, Pass HI, Carbone M, Yang H (2016). HMGB1 and Its Hyperacetylated Isoform are Sensitive and Specific Serum Biomarkers to Detect Asbestos Exposure and to Identify Mesothelioma Patients. Clin Cancer Res.

[20] Carbone M, Kanodia S, Chao A, Miller A, Wali A, Weissman D, Adjei A, Baumann F, Boffetta P, Buck B, de Perrot M, Dogan AU, Gavett S (2016). Consensus Report of the 2015 Weinman International Conference on Mesothelioma. J Thorac Oncol.

[21] Walter SD (2002). Properties of the summary receiver operating characteristic (SROC) curve for diagnostic test data. Stat Med.

[22] Leeflang MM (2014). Systematic reviews and meta-analyses of diagnostic test accuracy. Clin Microbiol Infect.

[23] Rutjes AW, Reitsma JB, Vandenbroucke JP, Glas AS, Bossuyt PM (2005). Case-control and two-gate designs in diagnostic accuracy studies. Clin Chem.

[24] Moher D, Liberati A, Tetzlaff J, Altman DG, Group P (2009). Preferred reporting items for systematic reviews and meta-analyses: the PRISMA statement. Ann Intern Med.

[25] Whiting PF, Rutjes AW, Westwood ME, Mallett S, Deeks JJ, Reitsma JB, Leeflang MM, Sterne JA, Bossuyt PM (2011). QUADAS-2: a revised tool for the quality assessment of diagnostic accuracy studies. Ann Intern Med.

[26] Reitsma JB, Glas AS, Rutjes AW, Scholten RJ, Bossuyt PM, Zwinderman AH (2005). Bivariate analysis of sensitivity and specificity produces informative summary measures in diagnostic reviews. J Clin Epidemiol.

[27] Han ZJ, Wu XD, Cheng JJ, Zhao SD, Gao MZ, Huang HY, Gu B, Ma P, Chen Y, Wang JH, Yang CJ, Yan ZH (2015). Diagnostic accuracy of natriuretic peptides for heart failure in patients with pleural effusion: A systematic review and updated meta-Analysis. PLoS One.

[28] Deeks JJ, Macaskill P, Irwig L (2005). The performance of tests of publication bias and other sample size effects in systematic reviews of diagnostic test accuracy was assessed. J Clin Epidemiol.

